# Effects of Jobelyn^®^ on Isoniazid-Induced Seizures, Biomarkers of Oxidative Stress and Glutamate Decarboxylase Activity in Mice

**DOI:** 10.32598/bcn.9.6.389

**Published:** 2018-11-03

**Authors:** Stephen Asehinde, Abayomi Ajayi, Adewale Bakre, Osarume Omorogbe, Adaeze Adebesin, Solomon Umukoro

**Affiliations:** 1. Department of Pharmacology and Therapeutics, College of Medicine, University of Ibadan, Ibadan, Nigeria.

**Keywords:** Sorghum bicolor, Seizures, Isoniazid, Glutamate decarboxylase, GABA

## Abstract

**Introduction:**

Isoniazid-induced seizure, often described as Status Epilepticus (SE), is an emergency condition characterized by repeated convulsive episodes that responds poorly to the currently available anticonvulsant drugs. The current study aimed at ascertaining the effect of Jobelyn^®^ (JB), an African dietary supplement, on seizures, altered oxidative stress, and glutamate decarboxylase activity induced by isoniazid in mice.

**Methods:**

A total of 6 mice received JB (10–50 mg/kg, PO), pyridoxine (300 mg/kg), diazepam (5 mg/kg), or distilled water (10 mL/kg) 30 minutes prior to the induction of SE with injection of isoniazid (300 mg/kg, IP). Thereafter, the mice were observed for the onset of convulsions for a period of two hours. Moreover, the effect of JB on Glutamate Decarboxylase (GAD) activity and biomarkers of oxidative stress (glutathione and malondialdehyde) was also evaluated in the brain homogenates of another set of isoniazid-treated mice.

**Results:**

JB (50 mg/kg, PO) prolonged the latency to convulsions, but could not prevent the occurrence of seizure episodes caused by isoniazid. Moreover, JB neither showed any protection against death nor delayed the latency to death caused by isoniazid. However, this dose of JB positively modulated the concentrations of malondialdehyde and glutathione in the brains of mice treated with isoniazid. The activity of GAD, the enzyme responsible for GABA synthesis, increased by JB, which suggested enhanced GABAergic neurotransmission.

**Conclusion:**

The current study findings suggest that JB prolongs the latency to convulsions, enhances GABAergic neurotransmission, and demonstrates anti-oxidative effect in isoniazid-treated mice.

## Highlights

- Jobelyn® (JB), an African dietary supplement, prolongs the latency to convulsions induced by isoniazid in mice, but fails to prevent the occurance of conculsions.
- JB neither protects against death nor delays the latency to death caused by isoniazid.
- JB inhibits oxidative stress in the brains of mice treated with isoniazid.
- JB increases the activity of glutamate decarboxylase, the enzyme responsible for GABA synthesis in isoniazid-treated mice.

## Plain Language Summary

Status Epilepticus (SE) is one of the major side effects of isoniazid, a first line drug used for the treatment of tuberculosis. The sustained seizure produced by isoniazid is due to the inhibition of glutamate decarboxylase, the enzyme that control the formation of GABA, a chemical that reduce the firing rate of nerve cells in the brain. The major sign of SE in patients with isoniazid poisoning is repeated convulsions, which often leads to the formation of toxic substances that damage the brain cells. Isoniazid-induced SE responds poorly to drugs currently used for the treatment of convulsion hence new agents should be sought. Jobelyn (JB) is an African food supplement obtained from Sorghum bicolor plant and widely used by the people of Western Nigeria to manage febrile seizures in children. To this end, we tested the ability of JB to prevent convulsion and death induced by isoniazid in experimental animals. Based on our findings, JB only delays the convulsion in animals but cannot prevent convulsion and death caused by isoniazid. JB decreased the amount of toxic substances produced by isoniazid in the brains of the animals. It also increased the activity of glutamate decarboxylase, which indicates the presence of more GABA in the brains of isoniazid-treated animals. However, despite the ability of JB to boost the activity of this enzyme and reduce the amount of toxic substances in brains of the animals, it still fails to prevent convulsion and death produced by this anti-TB drug.

## 1. Introduction

Status Epilepticus (SE) is a life-threatening medical condition often associated with persistent seizures and is recurrent by nature (Bassin, Smith, & Bleck, 2002; Brophy et al., 2012; Meierkord, Boon, & Engelsen, 2010). It accounts for about 3% to 5% of all cases of emergency admissions for seizure disorders. It also occurs in 2% to 16% of individuals with epileptic disorders (Hauser, 1990). The incidence of SE was estimated as 41 to 61 cases per 100000 patients per year (Bronstein et al., 2010; DeLorenzo, Towne, Pellock, & Ko, 1992).

Isoniazid, an anti-tuberculosis drug, induces SE by depleting brain level of Gamma-Aminobutyric Acid (GABA), a major inhibitory transmitter substance in the mammalian brain, through inhibition of pyridoxal-5-phosphate-dependent Glutamic Acid Decarboxylase (GAD) (Corda, Costa, & Guidtti, 1982; Uzman et al., 2013). Pyridoxal-5-phosphate is the active form of pyridoxine, a cofactor for GAD, and an enzyme required for GABA synthesis (Bassin et al., 2002; Brophy et al., 2012; Meierkord et al., 2010; Corda et al., 1982; Uzman et al., 2013). The decrease in GABA levels results in recurrent seizures that characterized SE (Corda et al., 1982; Uzman et al., 2013). Although isoniazid-induced seizure is known to respond poorly to currently available anticonvulsant drugs, intravenous diazepam is still used to control the seizure episodes in the absence of pyridoxine (Corda et al., 1982; Uzman et al., 2013; Tajender & Saluja, 2006; Romero & Kuczler, 1998).

On this basis, diazepam and pyridoxine, serving as reference drugs, were compared with the current study test substance. However, pyridoxine is reported as the only effective antidote for isoniazid toxicity and should be given in doses equivalent to the amounts of the ingested isoniazid in order to be effective (Uzman et al., 2013; Tajender & Saluja, 2006; Romero & Kuczler, 1998). However, when the quantity of the ingested isoniazid is unknown, intravenous administration of an initial dose of 5 g of pyridoxine is recommended in literature (Uzman et al., 2013; Tajender & Saluja, 2006). Although intravenous pyridoxine is relatively inexpensive, the immediate its sufficient amount is not always available in emergency (Tajender & Saluja, 2006; Romero & Kuczler, 1998). Thus, new compounds should be found that can serve as alternatives for isoniazid.

The recurrent seizures typifying SE are linked to increased oxidative stress that create a vicious cycle for neurodegeneration and manifestation of other neurological complications including memory deficits (Cicek et al., 2005; Adebayo, Oyagbemi, Akintunde, Akinyinka, & Arojojoye, 2012). The oxidative damage due to isoniazid is associated with the generation of reactive oxygen species, which initiate lipid peroxidative tissue damage (Cicek et al., 2005; Adebayo et al., 2012; Ahadpour et al., 2016; Cevik et al., 2012).

It also depletes endogenous antioxidant status of the cells thereby making the body organs more prone to the deleterious effect of oxidative stress (Adebayo et al., 2012; Ahadbpour et al., 2016; Cevik et al., 2012). However, the brain is more susceptible to oxidative stress damage due to high content of oxidizable fatty acids, high demand of oxygen, and low levels of antioxidant status (Moreira et al., 2008). Thus, the combination of isoniazid with a potent antioxidant agent such as Jobelyn (JB) (Benson et al., 2013) may help reduce brain damage and thus prevent the manifestations of various neurological complications associated with isoniazid neurotoxicity.

JB is a dietary supplement obtained from Sorghum bicolor L. (Gramineae family) that won international recognition for anemia, arthritis, and relief of stress (Benson et al., 2013; Okochi, Okpuzor, Okubena, & Awoyemi, 2003). The most active compounds in JB include apigenin, luteolin, and naringenin, which possess various biological activi ties(Benson et al., 2013; Umukoro, Oluwole, Eduviere, Adrian, & Ajayi, 2015). Previous investigations revealed that JB has potent antioxidant and anti-inflammatory properties (Benson et al., 2013; Umukoro et al., 2015). Moreover, JB has been listed as one of the herbal remedies used by the populace to manage febrile seizures (Oshikoya, Senbanjo, Njokanma, & Soipe, 2008).

It was previously established that JB prolonged the latency to convulsions induced by pentylenetetrazole in mice (Umukoro, Omogbiya, & Eduviere, 2013), which suggests its possible efficacy in controlling convulsive episodes (Umukoro et al., 2013). However, the current study aimed at investigating the possible protective effect of JB against isoniazid-induced convulsion, altered oxidative stress, and Glutamate Decarboxylase (GAD) activity in mice.

## 2. Methods

### 2.1. Laboratory animals

Male Swiss mice (weighed 20–22 g) used in the study were purchased from the Central Animal House, University of Ibadan, Nigeria. They were housed in plastic perplex cages at room temperature, had free access to rodent pellet diet, and water. The experimental procedure was conducted in accordance with the National Institute of Health (NIH) Guidelines for the Care and Use of Laboratory Animals.

### 2.2. Drugs and chemicals

JB (Forever Product Lagos, Nigeria), isoniazid (Hamex Medica Ltd., UK), diazepam (Swipha Pharma, UK), Tri-Chloroacetic Acid-TCA (Burgoyne Burbidges & Co., Mumbai, India), Thiobarbituric Acid (TBA) (Sigma, Germany), 5,5′-Dithio-Bis(2-Nitrobenzoic acid) (DTNB) (Sigma Aldrich, Germany), Glutamate (Sigma Aldrich, Germany), and Pyridoxine (Pauco Pharamceutical Industry, Nigeria) were used in the study. JB and other drugs were dissolved in distilled water immediately before use. The doses of JB were selected based on previous studies (Umukoro et al., 2013).

### 2.3. Effect of Jobelyn on the onset of seizure

The effect of JB on isoniazid-induced convulsion was assessed as described (Corda et al., 1982). The animals were divided into different treatment groups (n=6) and were given distilled water (control, 10 mL/kg), diazepam (5 mg/kg), pyridoxine (300 mg/kg) or JB (10, 25, 50 mg/kg) orally. Thirty minutes later, the mice were given isoniazid (300 mg/kg, IP) and were observed for two hours for latency to convulsion and death.

### 2.4. Biochemical studies

New set of animals were pretreated orally with distilled water (control, 10 mL/kg), JB (10, 25, 50 mg/kg), diazepam (5 mg/kg) or pyridoxine (300 mg/kg) 30 minutes prior to intraperitoneal injection of isoniazid (300 mg/kg). Thereafter, the mice were euthanized 30 minutes post-isoniazid injection through cervical dislocation under ether anesthesia. The brains were removed, weighed, and kept in 10% w/v phosphate buffer (0.1M; pH 7.4). The whole brains were then homogenized in 10% w/v phosphate buffer and the supernatants were used for the biochemical studies.

#### 2.4.1. Determination of glutathione concentration

The brain level of reduced GSH was determined as described (Moron, Depierre, & Mannervik, 1979). Briefly, the supernatant (0.4 mL) of the brain tissue was added to 20% trichloroacetic acid (0.4 mL) and then, centrifuged at 10000 rpm for 20 min at 4°C. This mixture (0.25 mL) was added to 2 mL of 0.6 mM DTNB and the final volume was made up to 3 mL with phosphate buffer (0.2M, pH 8.0). Next, the absorbance was read at 412 nm against blank reagent using a spectrophotometer. The brain levels of reduced GSH were expressed as μM/g tissue.

#### 2.4.2. Estimation of brain concentration of malondialdehyde

MDA concentrations in the brain supernatants were determined as previously described (Ohkawa, Ohishi, & Yagi, 1979). Briefly, distilled water (0.5 mL) and 10% TCA (1.0 mL) were added to 0.5 mL of each homogenate of the brain tissue. This mixture was then centrifuged at 3000 rpm for 10 minutes and 0.1 mL of thiobarbituric acid (0.375%) was added to 0.4 mL of the supernatant. This mixture was then incubated in a water bath at 80°C for 40 minutes. After cooling, the absorbance of the supernatant was read at 532 nm using a spectrophotometer. The brain levels of MDA were expressed as μM/g tissue.

#### 2.4.3. Determination of GAD activity

Brain GAD activity was determined as earlier described (Cozzani, 1970). The brain supernatant (1 mL) was adjusted to pH 7 and then incubated at 37°C for a period of 5 min. Thereafter, the reaction started by adding 100 μL glutamate (10 mM) and decarboxylation of glutamate was measured at 340 nm against a blank including all the components except glutamate using spectrophotometer (Cozzani, 1970).

### 2.5. Statistical analysis

The obtained data were analyzed with Graph Pad Prism software, version 4.0 and expressed as Mean±standard Error of the Mean±SEM. Statistical analysis was conducted using 1-way ANOVA followed by the Newman-Keuls posthoc test. P values less than 0.05 were considered statistically significant.

## 3. Results

### 3.1 Jobelyn prolongs latency to seizure

As shown in Table 1, intraperitoneal injection of isoniazid (300 mg/kg) produced 100% convulsion and death, as all the animals convulsed and died within the experimental period. However, JB (50 mg/kg, PO) prolonged the latency to seizure episodes (P<0.05), but failed to prevent occurrence of seizure episodes or death (Table 1). In contrast, diazepam but not pyridoxine offered 30% protection against convulsions and death in isoniazid-treated mice (Table 1).

**Table 1:** Effect of Jobelyn, Diazepam, and Pyridoxine on Isoniazid-induced seizure episodes in mice

**Treatment**	**Onset of Seizure (Min)**	**Seizure (%)**	**Latency to Death (Min)**	**Death (%)**
Control	48.67±1.687	100	53.83±1.302	100
JB (10 mg/kg)	48.17±1.493	100	51.50±3.085	100
JB (25 mg/kg)	52.67±3.518	100	58.50±3.008	100
JB (50 mg/kg)	62.33±3.870^*^	100	62.50±4.552	100
DZ (5 mg/kg)	85.17±2.072^*^	70	88.83±10.95^*^	70
Pyridoxine (300 mg/kg)	35.83±2.496^*^	100	35.67±2.565	100

Values are expressed as Mean±SEM for 5 animals per group.

* P<0.05 compared with controls (ANOVA followed by the Newman-Keuls test).

Abbreviations: JB: Jobelyn; DZ: Diazepam

### 3.2. Jobelyn reduces isoniazid-induced oxidative stress in mouse brain

**Figure 1:**
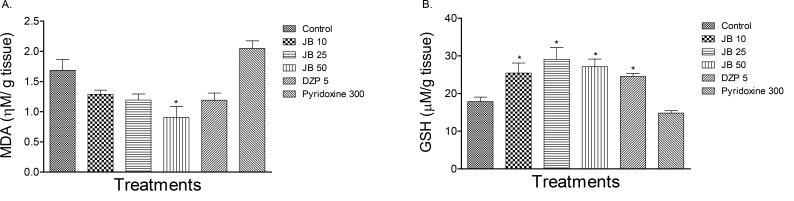
Effect of Jobelyn (JB), Diazepam (DZ) and pyridoxine on the brain levels of Malondialdehyde (MDA, panel A) and Glutathione (GSH, panel B) in isoniazid-treated mice Each column represents Mean±SEM for 6 animals per group. P<0.05 compared to control (ANOVA followed by Newman Keuls test).

Figures 1 A and B showed the effect of JB on the brain MDA and GSH levels in isoniazid-treated mice. Isoniazid (300 mg/kg, IP) altered the concentrations of these biomarkers of oxidative stress relative to those of the control (P<0.05). As presented in Figure 1 A and B, JB (10, 25, 50 mg/kg, PO) or diazepam (5 mg/kg, PO) attenuated the altered MDA and GSH levels in the brains of mice treated with isoniazid (P<0.05).

### 3.3. Effect of Jobelyn on GAD activity in mice brains

**Figure 2:**
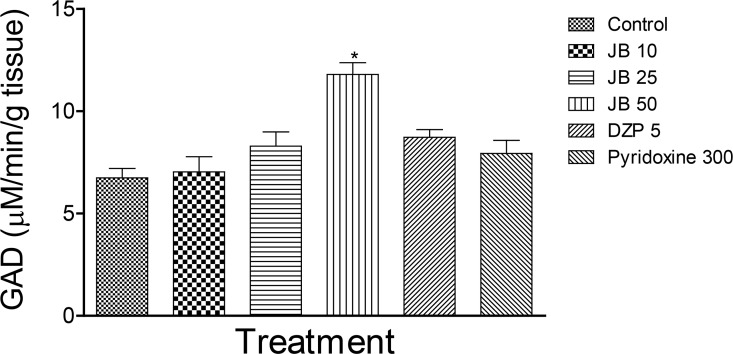
Effect of Jobelyn (JB), Diazepam (DZ), and pyridoxine on Glutamate Decarboxylase (GAD) activity Each column represents mean±SEM for six animals per group. P<0.05 compared with controls (ANOVA followed by the Newman-Keuls test).

The effect of JB on GAD activity in mice with isoniazid-induced seizure is shown in Figure 2. JB (50 mg/kg, PO), but not diazepam or pyridoxine, significantly elevated the activity of GAD in isoniazid-treated mice.

## 4. Discussion

The results of the current study revealed that JB did not prevent the occurrence of seizures induced by isoniazid in mice. However, the highest dose of JB significantly delayed the onset of seizures. In contrast, diazepam, but not pyridoxine, offered 30% protection against convulsions and death in isoniazid-treated mice. Moreover, JB and diazepam decreased the brain MDA level and increased the GSH content suggesting antioxidant properties. However, JB, but not diazepam or pyridoxine, produced a significant increase in GAD activity, which suggested its ability to elevate GABA levels in the brains of mice treated with isoniazid.

SE is one of the key features of isoniazid poisoning or overdose and refractory to conventional anticonvulsant drugs (Bassin et al., 2002; Brophy et al., 2012; Meierkord et al., 2010). SE induced by isoniazid is related to inhibition of GAD, an enzyme required for GABA synthesis (Bassin et al., 2002; Bronstein et al., 2010; Corda et al., 1982; Cevik et al., 2012). Thus, the depletion of brain GABA levels is associated with continuous seizures observed in animals exposed to high doses of isoniazid. Treatment approach for isoniazid toxicity involves intravenous injection of pyridoxine, an essential cofactor required for GABA synthesis (Bassin et al., 2002; Brophy et al., 2012; Meierkord et al., 2010). However, the uncertainty of the amount of INH ingested and its immediate availability are often the major limitations to the usefulness of pyridoxine in managing isoniazid toxicity in clinical settings (Minns, Ghafouri, & Clark, 2010).

In the current study, the reasons why pyridoxine unlike diazepam could neither prevent nor delay INH-induced convulsions in mice were not apparent in the current investigation except that previous studies showed that pyridoxine was ineffective against isoniazid-induced seizures in mice (Bonner, Peterson, & Weir, 1999). Moreover, the reason (s) of pyridoxine protection of rats but not mice against convulsions induced by isoniazid was not also obvious from the study of Bonner et al. (1999).

Previous studies show that anticonvulsant effect of a novel agent does not absolutely depend on the prevention of convulsions, but on its ability to prolong the latency to seizures (Kendall, Fox, & Enna, 1981). Moreover, compounds that only delay the latency to convulsions block the spread of seizures in an epileptic brain (Corda et al., 1982). Thus, the finding that JB prolonged the latency to isoniazid-induced convulsions suggests anticonvulsant potentials in mice. The finding that JB also increased brain activity of GAD, an enzyme responsible for the synthesis of GABA, suggests its ability to elevate GABA levels in the brain. It is instructive to note that reduced brain GABA concentrations are implicated in the pathophysiology of convulsion (McNamara, 1994). Thus, the increase of GABA levels in the brain is one of the major therapeutic approaches in treating patients with seizures (McNamara, 1994). Nevertheless, more studies are necessary to establish the role of GAD in the anticonvulsant potential of JB in isoniazid-induced convulsions in mice.

Increased oxidative stress and subsequent manifestation of other neurological complications are associated with isoniazid toxicity (Cicek et al., 2005; Adebayo et al., 2012; Ahadpour et al., 2016; Cevik et al., 2012). Isoniazid-induced oxidative tissue damage is related to the formation of reactive oxygen species, which in turn initiate lipid peroxidation in most organs of the body, especially the liver and the brain (Cicek et al., 2005; Adebayo et al., 2012; Ahadpour et al., 2016; Cevik et al., 2012).

It also depletes endogenous antioxidant status of the cells, thereby exposing body organs to the deleterious effect of oxidative stress (Adebayo et al., 2012; Ahadpour et al., 2016; Cevik et al., 2012). However, the brain cells are more prone to deleterious effect of oxidative stress due to high content of oxidizable fatty acids, high oxygen demand, and low levels of antioxidant molecules (Moreira et al., 2008).

In recent years, a great number of natural products are sought from plants to mitigate oxidative stress-mediated pathologies (Mahmoud, Germoush & Soliman, 2014). For example, berberine, an alkaloid found in a variety of plants protects the liver against isoniazid-induced hepatotoxicity via inhibition of oxidative stress (Mahmoud et al., 2014). In addition, several phytochemicals demonstrate neuroprotection and are still being evaluated as potential therapeutic agents for neurological diseases (Natalie, Kelsey, Heather, Wilkins, & Linseman, 2010). Moreover, previous preclinical investigations revealed that JB had antioxidant and neuroprotective properties in various experimental models (Benson et al., 2013; Oyinbo, Dare, Avwioro, & Igbigbi, 2015).

Meanwhile, JB also demonstrated antioxidant property in the mentioned studies as it attenuated isoniazid-induced increase in brain MDA level, a well-known marker of oxidative stress implicated in lipid peroxidative tissue damage. This finding was in agreement with the reports of previous studies (Oyinbo et al., 2015) and further suggested that JB supplementation may protect the neurons against isoniazid-induced neurotoxicity. On the other hand, GSH content in brain significantly decreased in isoniazid-treated mice.

Glutathione is a key endogenous antioxidant agent that protects cellular constituents against the harmful effects of oxidative stress by scavenging ROS (Franco, Schonveld, Pappa, & Panayiotidis, 2007). However, pretreatment with JB inhibited depletion of GSH suggesting its ability to scavenge ROS generated by isoniazid in mice brains. Thus, the antioxidant effect exhibited by JB suggests its potential benefits to prevent neurological complications associated with isoniazid therapy.

The results of the current study suggested that increased GAD activity might contribute to the ability of JB to prolong the latency to convulsions induced by isoniazid. The current study also suggests that JB supplementation might be useful as a potential antioxidant agent in preventing oxidative stress-induced neurological complications associated with anti-tuberculosis therapy with isoniazid.

## Ethical Considerations

### Compliance with ethical guidelines

The experimental procedures utilized in this study were in accordance with the National Institute of Health (NIH) guidelines for the care and use of Laboratory animals.
